# Local atmospheric response to warm mesoscale ocean eddies in the Kuroshio–Oyashio Confluence region

**DOI:** 10.1038/s41598-017-12206-9

**Published:** 2017-09-19

**Authors:** Shusaku Sugimoto, Kenji Aono, Shin Fukui

**Affiliations:** 1 Frontier Research Institute for Interdisciplinary Sciences, Tohoku University, Sendai, 980-8578 Japan; 2 Department of Geophysics, Graduate School of Science, Tohoku University, Sendai, 980-8578 Japan

## Abstract

In the extratropical regions, surface winds enhance upward heat release from the ocean to atmosphere, resulting in cold surface ocean: surface ocean temperature is negatively correlated with upward heat flux. However, in the western boundary currents and eddy-rich regions, the warmer surface waters compared to surrounding waters enhance upward heat release–a positive correlation between upward heat release and surface ocean temperature, implying that the ocean drives the atmosphere. The atmospheric response to warm mesoscale ocean eddies with a horizontal extent of a few hundred kilometers remains unclear because of a lack of observations. By conducting regional atmospheric model experiments, we show that, in the Kuroshio–Oyashio Confluence region, wintertime warm eddies heat the marine atmospheric boundary layer (MABL), and accelerate westerly winds in the near-surface atmosphere via the vertical mixing effect, leading to wind convergence around the eastern edge of eddies. The warm-eddy-induced convergence forms local ascending motion where convective precipitation is enhanced, providing diabatic heating to the atmosphere above MABL. Our results indicate that warm eddies affect not only near-surface atmosphere but also free atmosphere, and possibly synoptic atmospheric variability. A detailed understanding of warm eddy–atmosphere interaction is necessary to improve in weather and climate projections.

## Introduction

Vigorous heat related to the turbulent heat flux (THF; sum of latent and sensible heat fluxes) is released from the ocean to the atmosphere during winter in the Kuroshio–Oyashio Confluence (KOC) region; the value and variance are one of the largest in the world’s oceans (Supplementary Fig. 1). Observational studies have shown that the sea surface temperature (SST) is primarily responsible for the upward THF release in the KOC region^1–4^—a huge amount of heat is released where there are positive SST anomalies. Recent studies of the KOC region using atmospheric reanalysis dataset have reported that an increase in THF is associated with warming of the marine atmospheric boundary layer (MABL) and enhancement of surface winds attributable to the vertical mixing effect^5,6^. In this process, warm SST decreases the near-surface static stability and thereby enhances downward transport of wind momentum to locally accelerate surface winds^7,8^. These findings indicate that the ocean can force the overlying atmosphere in the KOC region.

The KOC region is one of the regions of highest eddy kinetic energy in the world’s oceans^9,10^, due to (1) perturbations of the Kuroshio Extension (KE) (i.e., meanders of the KE)^11,12^; (2) warm (anticyclonic) mesoscale ocean eddies with a horizontal extent of a few hundred kilometers and a thickness of a few hundred meters, which pinch off from the KE in a northward direction^13,14^ (Supplementary Fig. 2); and (3) other mesoscale perturbations. High-spatial-resolution satellite measurements revealed that SST anomalies in the KOC region are formed through pinched-off warm mesoscale ocean eddies^2,3^, suggesting that warm eddies are essential to understanding atmosphere–ocean interaction in the region.

Despite the importance of warm mesoscale ocean eddies in the atmosphere–ocean interaction system, previous studies have been constrained by several shortcomings: the coarse horizontal resolution of atmospheric reanalysis data limits our ability to robustly detect the effects of warm eddies on the atmosphere; insufficient *in situ* data has hindered investigation of the warm-eddy-induced imprints on the atmospheric field; and satellite measurements have been unable to capture vertical atmospheric structures over the warm eddies. In this study, to reveal the extent to which the warm mesoscale ocean eddies influence the overlying atmosphere, we conduct regional atmospheric model experiments using the high-resolution Japan Meteorological Agency nonhydrostatic model (JMA-NHM)^15,16^ with 27 km horizontal grid spacing.

## Results

**Figure 1:**
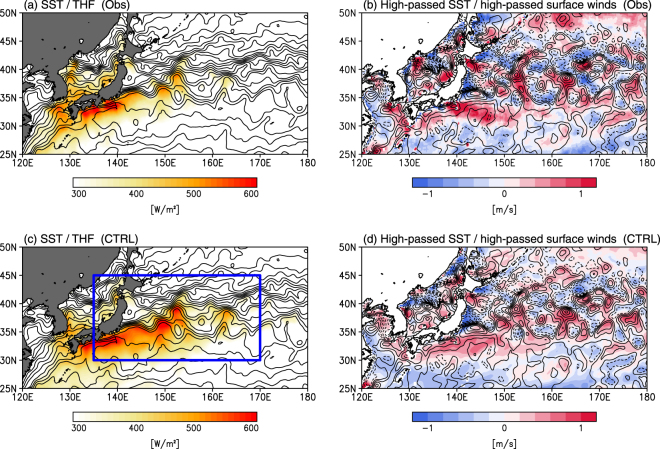
Satellite-derived observations *versus* CTRL run. (**a**) Upward THF from J-OFURO3 (color in W m^−2^) and SST from OISST (contours with an interval of 1 °C) averaged over December 2003. (**b**) SST from OISST (contours with an interval of 1 °C) and 10 m wind speed (color shading, in m s^−1^) from CCMP averaged over December 2003, high pass filtered using a 5° × 5° boxcar filter. (**c,d**) Same as (**a,b**), but for CTRL run, in the same month (see Methods). In (**c**), the blue rectangle represents an area shown in Fig. 2a,b. All plots are generated with GrADSv2.1.0 (http://cola.gmu.edu/grads/grads.php).

To detect imprints of warm mesoscale ocean eddies on the atmosphere, we conduct two sets of experiments by imposing different SST boundary conditions: the control (CTRL) run, where the observed SST field for 2003 is prescribed, with no warm eddies in the KOC region^14^, and the EDDY run, in which positive anomalies (Supplementary Fig. 3) are added to the SST field of CTRL to represent warm eddies (see Methods). The CTRL run successfully captures the large THF release (greater than 400 W m^−2^) in a narrow (~500 km) zonal band south of the SST front along the KE northern boundary (Fig. 1c), as observed from satellites (Fig. 1a); the spatial correlation coefficient between CTRL-THF (Fig. 1c) and satellite-derived THF (Fig. 1a) is high (*R* = 0.67). High-spatial-resolution satellite measurements indicate a local near-surface atmospheric response to the mesoscale SST^17–20^. Figure 1b shows a striking correspondence between the mesoscale SST and surface winds in observations. The CTRL run successfully reproduces the mesoscale relationship; high-passed surface wind in CTRL (Fig. 1d) is spatially correlated (*R* = 0.89) with surface wind observations (Fig. 1b). These provide confidence that JMA-NHM is capable of successfully reproducing the ocean mesoscale imprints on the overlying atmosphere. In the following paragraphs, we show results averaged over December, and averaged over an ensemble.

**Figure 2:**
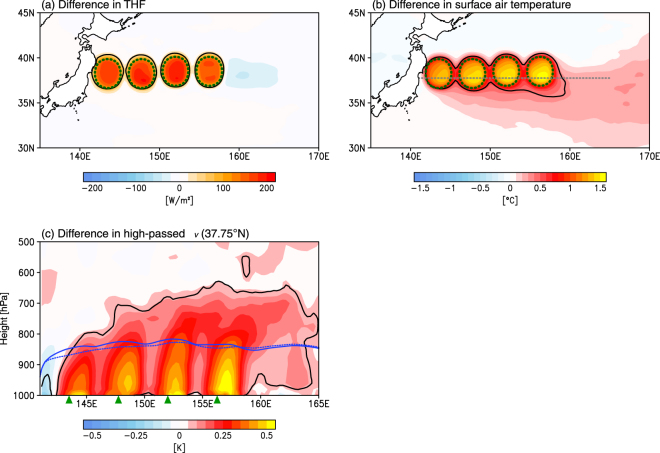
THF and air temperature response to warm ocean eddies. (**a**) Difference in THF (W m^−2^) between EDDY and CTRL runs (EDDY − CTRL). Black solid lines represent statistically significant regions exceeding 90% confidence level. Green dashed lines schematically indicate the position of warm ocean eddies in EDDY run (see Supplementary Fig. 3). (**b**) Same as (**a**), but for surface air temperature difference (°C). The gray dashed line is along 37.75°N. (**c**) Same as (**a**), but for a cross-section of high-pass-filtered virtual potential temperature difference (K) along 37.75°N. Green triangles at the bottom of the panel indicate latitudinal positions of warm ocean eddy centers in EDDY run. The blue solid and dashed lines represent the MABL height in EDDY and CTRL, respectively. All plots are generated with GrADSv2.1.0 (http://cola.gmu.edu/grads/grads.php).

A comparison between the EDDY and CTRL runs shows local THF increases over the warm mesoscale ocean eddies (Fig. 2a), with the value increasing by 180 W m^−2^ in the EDDY run. The large release of THF over eddies results in a warming of the overlying surface air of about 1.0–1.5 °C (Fig. 2b). We examine the upward influence of warm ocean eddies by showing the difference in high-pass-filtered virtual potential temperature, along 37.75°N, where a marked difference in surface air temperature can be seen (Fig. 2b), between the two runs (Fig. 2c). A 5° × 5° boxcar filter is used to highlight the warm-eddy-related signals. Significant warming extends not only into the near-surface atmosphere but also throughout the MABL, penetrating into the free atmosphere above the MABL, towards the eastern eddies, and reaching the 700 hPa level.

**Figure 3:**
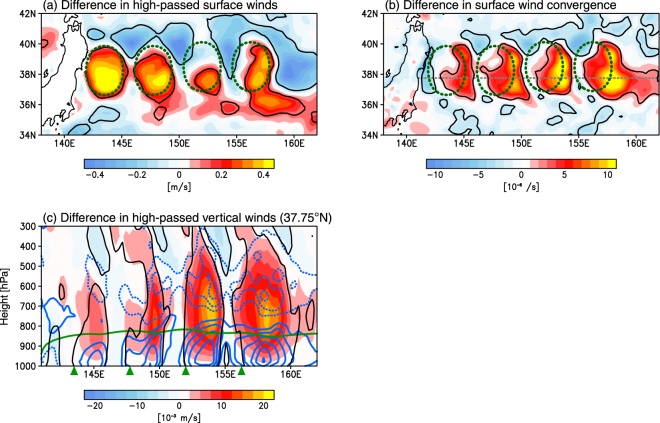
Wind response to warm ocean eddies. (**a,b**) Same as Fig. 2a, but for (**a**) high-pass-filtered surface winds (m s^−1^) and (**b**) surface wind convergence (10^−6^ s^−1^). (**c**) Same as Fig. 2c, but for high-pass-filtered upward vertical winds (color shading, in 10^−3^ m s^−1^). Blue solid and dashed contours represent wind convergence and divergence, respectively (the contour interval is 2 × 10^−6^ s^−1^ and zero contours are omitted). The green line indicates the MABL height in EDDY run. All plots are generated with GrADSv2.1.0 (http://cola.gmu.edu/grads/grads.php).

Winds within the MABL are locally enhanced over the warm eddies in the KOC region (Fig. 3a and Supplementary Fig. 4), and are stronger closer to the sea surface (Supplementary Fig. 4). Similar to winds, eddy momentum diffusion coefficients are larger in the MABL over warm eddies (Supplementary Fig. 4), indicating that enhanced surface winds are attributable to the downward transfer of large momentum from the upper MABL, via the vertical mixing effect. The vertical mixing brings westerly components aloft down to the surface, owing to the presence of monsoonal westerly winds aloft in winter. This results in an acceleration of surface westerly winds and consequent wind convergence around the eastern edge of eddies (Fig. 3b and c). The surface wind convergence gives local ascent with considerable vertical extent; the upward wind velocity is largest just above the MABL between the 800 and 700 hPa levels (Fig. 3c). The horizontal distribution in EDDY run shows collocation of strong upward wind velocity at these levels and enhanced surface wind convergence (Supplementary Fig. 5). The ascending motion structure deepens towards the eastern eddies where surface wind convergence is stronger (Fig. 3c). Indeed, the collocation of ascending motion and surface wind convergence is clearly visible at the 500 hPa level above the eastern eddies (Supplementary Fig. 5).

**Figure 4:**
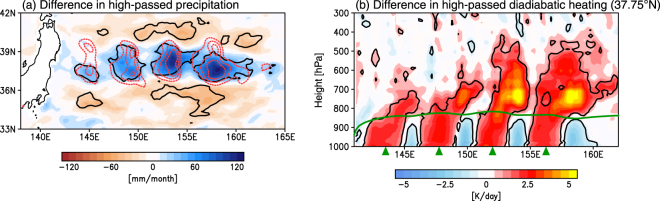
Precipitation response to warm ocean eddies. (**a**) Same as Fig. 2b, but for high-pass-filtered precipitation (mm month^−1^). (**b**) Same as Fig. 2c, but for high-pass-filtered diabatic heating rate (K day^−1^), calculated following Yanai^41,42^. All plots are generated with GrADSv2.1.0 (http://cola.gmu.edu/grads/grads.php).

Furthermore, our experiments reveal a collocation of enhanced precipitation and surface wind convergence (Fig. 4a); the precipitation increase attributable to the warm eddies is about 100 mm month^−1^, which corresponds to 30–50% of precipitation in the unfiltered CTRL run. Enhanced precipitation is induced mostly by convection (Supplementary Fig. 6). It is therefore expected that a large amount of precipitation affects the air temperature through diabatic heating. Figure 4b shows the difference, along 37.75°N, in the diabatic heating rate between the two runs, revealing a different response in and above the MABL. In the MABL, there is strong diabatic heating over the warm eddies (Fig. 4b and Supplementary Fig. 7), indicating that the warm eddies heat the MABL directly and locally. In contrast, the strong heating shifts eastward above the MABL and is largest just above the MABL top—between the 800 and 700 hPa levels (Fig. 4b)—and collocated with the strongest ascending motion (Fig. 2c). Indeed, the large diabatic heating at these levels (Supplementary Fig. 7) is collocated with the enhanced surface wind convergence, forming strong ascending motion. The diabatic heating also has a deeper structure over eddies located in the eastern part of the KOC region compared with those in its western part (Fig. 4b), as well as in the ascending motion (Fig. 3c), which roughly overlaps the warming area above the MABL (Fig. 2c). This close covariation in space is strongly indicative of an active role of warm eddies in warming the atmosphere.

## Summary and Discussion

Our regional atmospheric model experiments reveal that wintertime warm mesoscale ocean eddies in the KOC region heat the MABL directly and locally, through a large upward release of THF. This accelerates westerly winds in the near-surface atmosphere by the vertical mixing effect—owing to the presence of monsoonal westerly winds aloft during winter—leading to surface wind convergence around the eastern edge of the eddies. The eddy-induced convergence gives local ascending motion extending to the middle/upper troposphere; here, convective precipitation is enhanced, providing strong diabatic heating to the atmosphere above the MABL.

Recent studies have shown that momentum diffusion is large in the MABL over subtropical warm ocean eddies in the South Atlantic^21^ and Indian Ocean^22^, which is consistent with our results. However, past studies also reported that the precipitation anomaly increase not above the near surface wind convergence zone but above the maximum SST anomaly of warm eddies in the Southern Ocean^23^. This contrasts with our result that the precipitation anomaly occurs above the convergence zone, slightly downstream of the maximum SST anomaly. This implies that the mechanisms through which warm eddies affect the atmosphere are not necessarily identical among ocean basins.

Vigorous release of heat associated with warm ocean eddies in the KOC region can explosively develop extratropical cyclones^24^; these can be similar in strength to tropical cyclones and can lead to severe weather disasters caused by heavy rainfall/snowfall, strong winds, and high waves. In our results, it looks like the effect of eddies on the atmosphere is increasing downstream even though the SST anomalies are the same for all idealized eddies, implying regional difference of eddy effects. Establishment of a general and regional framework that describes how warm eddies drive the atmosphere could greatly improve weather forecasting skill.

The global subtropical western boundary currents and their extensions, from which warm eddies are pinched off, show an accelerated warming over the 20th century that far exceeds the globally averaged surface warming rate^25^. Unraveling the processes through which warm eddies associated with changing western boundary current systems influence synoptic atmospheric variability require organized long-term observational and modeling efforts. This will enable more accurately predictions of the synoptic atmospheric variability response to future climate change.

## Methods

### Observational data

We used the following satellite-derived products: the NOAA optimum interpolation SST (OISST) product^26^ for 16 November to 31 December 2003 on a 0.25° grid; the THF product of the Japanese Ocean Flux Data Sets with Use of Remote Sensing Observations version 3 (J-OFURO3) (https://j-ofuro.scc.u-tokai.ac.jp) for 1 to 31 December 2003 on a 0.25° grid; and the Cross-Calibrated Multi-Platform (CCMP) ocean surface wind velocity^27^ for 1 to 31 December 2003 on a 0.25° grid. Detailed information on each dataset is provided in the corresponding reference.

### JMA-NHM experiments

The regional atmospheric model used in this study is JMA-NHM^15,16^, which is designed to serve both weather forecasting and atmospheric research needs, and has been used in a variety of regional climate studies. The domain covers the North Pacific and western North Atlantic from 15°N to 70°N and 110°E to 320°E in the Mercator projection, with 27 km horizontal grid spacing. There are 51 vertical levels with realistic topography; 19 levels are placed below 2 km high in order to finely resolve the MABL. We used the Mellor–Yamada–Nakanishi–Niino level-3 planetary boundary layer scheme^28^, the Beljaars–Holtslag flux and bulk coefficient scheme^29^, and the Kain–Fritsch convective parameterization scheme^30,31^ for convective processes.

We specifically focus on the months of December, when THF reaches its maximum value in the KOC region (Supplementary Fig. 8). We conducted two sets of experiments by imposing different SST boundary conditions. The first is the control (CTRL) run, in which we prescribe daily SST from OISST for 16 November to 31 December 2003, when no warm eddies were pinched off the KE in the KOC region^14^, and with a near-neutral phase of natural variability due to El Niño and the Pacific Decadal Oscillation. The second experiment (EDDY run) is conducted by adding positive anomalies—each representing a warm eddy—to the daily SST field of CTRL; we set four warm eddies, each with an SST anomaly of 3 °C at their centers and a horizontal extent of about 300 km (Supplementary Fig. 3). This differs from the traditional method of using observed SST data and spatially smoothed data as the boundary condition^32–39^, an approach that is designed to investigate the total influence of SST frontal structure and mesoscale perturbations, including the pinched-off warm eddies, on the atmosphere. Our experimental design allows us to reveal the atmospheric response to the pinched-off warm eddies alone. The CTRL and EDDY experiments each consist of 10 NHM simulations, in which the initial and boundary conditions were taken from the Japanese 55-year Reanalysis Project (JRA-55) dataset^40^ on the same day but for different years, i.e., 2000, 2001, 2002, 2003, 2004, 2005, 2006, 2006, 2008, 2009, respectively. The simulation period extends from 16 November to 31 December. A period between 16 November to the end of that month is a spin-up phase and then December is analyzed. All results show an average over December and an average over the ensemble. It is worth emphasizing that in this regional modeling approach the initial and lateral boundary conditions are identical in the two experiments.

### Data availability

The data are available from the corresponding author upon reasonable request.

## Electronic supplementary material

Supplementary Information
