# Supplementary material for: Local atmospheric response to warm mesoscale ocean eddies in the Kuroshio–Oyashio Confluence region
Source: Sci Rep. 2017 Sep 19;7:11871. doi: 10.1038/s41598-017-12206-9 (PMC5605681; doi:10.1038/s41598-017-12206-9)
Supplement: Supplementary file 1 — Supplementary Information [file 41598_2017_12206_MOESM1_ESM.pdf]

Supplementary Information

Local atmospheric response to warm mesoscale ocean eddies in the Kuroshio–Oyashio Confluence region

Shusaku Sugimoto[1]*, Kenji Aono[2], Shin Fukui[2]

1. Frontier Research Institute for Interdisciplinary Sciences, Tohoku University, Sendai 980-8578, Japan
2. Department of Geophysics, Graduate School of Science, Tohoku University, Sendai 980-8578, Japan

*e-mail: sugimoto@pol.gp.tohoku.ac.jp

This PDF file includes:

Supplementary Figures 1 to 8

References used in Supplementary Information

43. Tomita, H. *et al*. An assessment of surface heat fluxes from J-OFURO2 at the KEO and JKEO sites. *J Geophys Res* **115,** C03018, doi:10.1029/2009JC005545 (2010).

44. Kubota, M., Iwasaka, N., Kizu, S., Konda, M. & Kutsuwada, K. Japanese ocean flux data sets with use of remote sensing observations (J-OFURO). *J Oceanogr* **58,** 213–225 (2002).

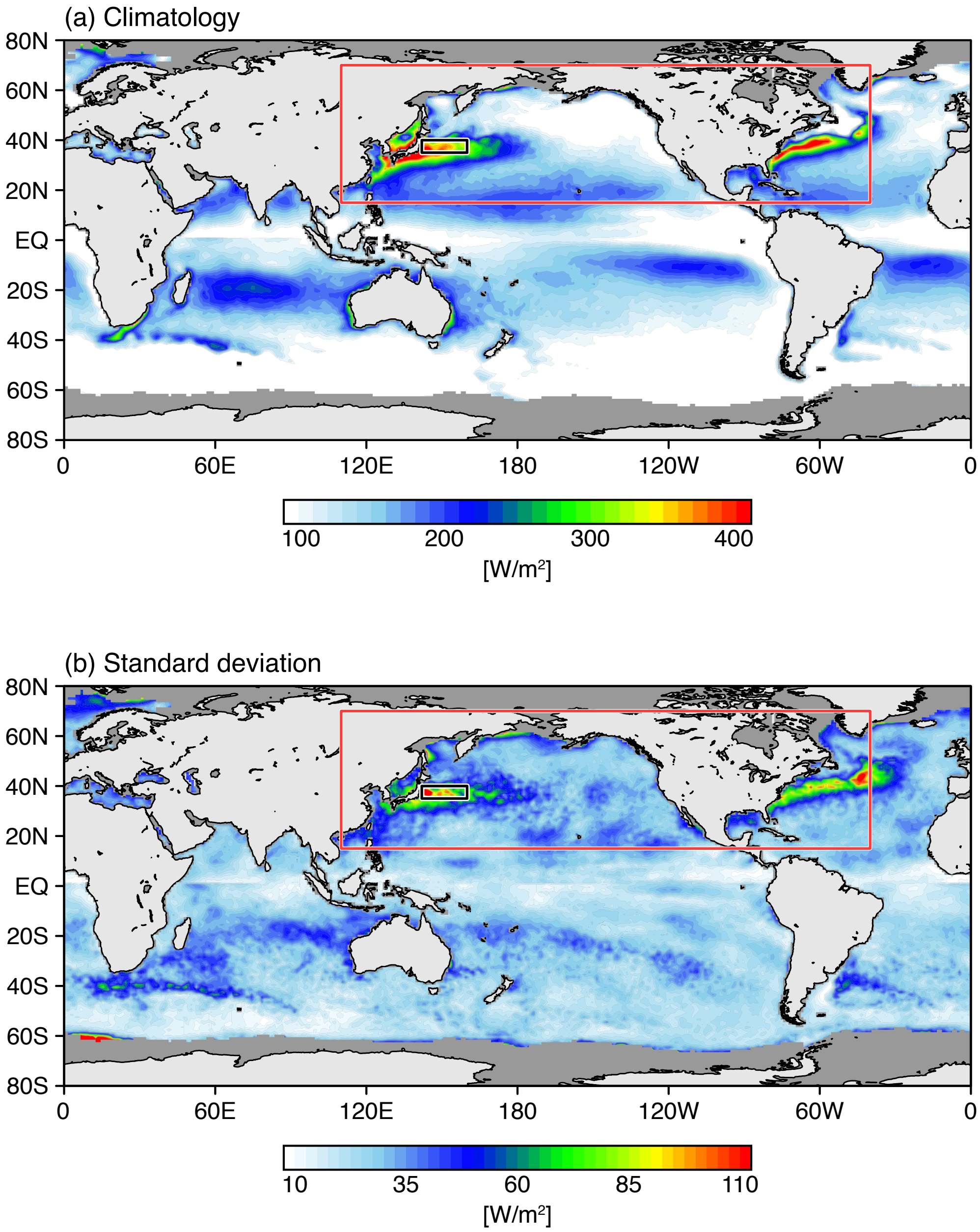

Supplementary Figure 1. Spatial features of satellite-derived THF. (**a**) Climatology and (**b**) standard deviation of the mean upward turbulent heat flux (THF) (W m^{-2}) for the Northern Hemisphere in December and the Southern Hemisphere in June, from Japanese Ocean Flux Data Sets with Use of Remote Sensing Observations version 2 (J-OFURO2, ref. 43, 44) on a 1° grid. The black and red rectangles represent the Kuroshio–Oyashio Confluence (KOC) region (142°E–160°E, 35°N–40°N) and the JMA-NHM calculation domain (110°E–320°E, 15°N–70°N), respectively. All plots are generated with GrADSv2.1.0 (http://cola.gmu.edu/grads/grads.php).

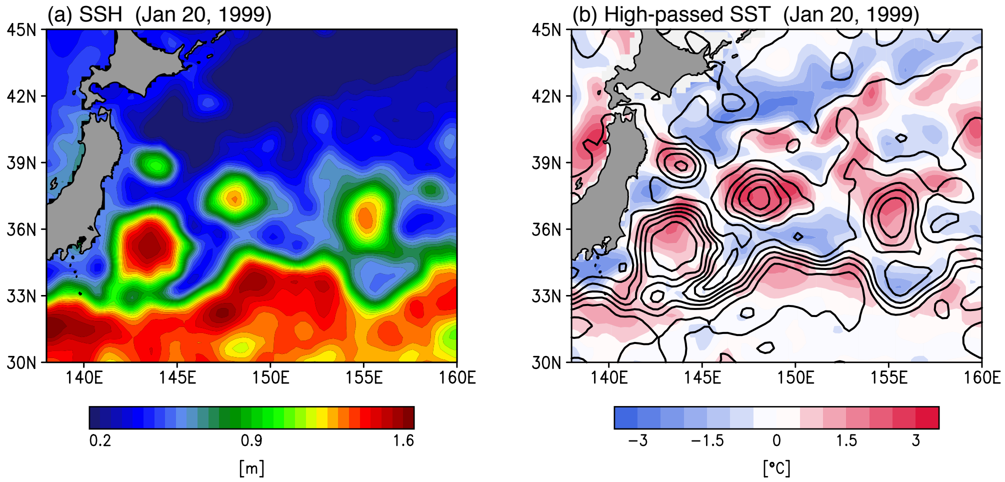

Supplementary Figure 2. Warm ocean eddies pinched northward off the Kuroshio Extension. Snap shots for 20 January 1999 of (**a**) sea surface height (SSH) (m) on a 0.25° grid, distributed by AVISO (http://www.aviso.altimetry.fr) and (**b**) sea surface temperature (SST) (°C) from the NOAA optimum interpolation SST product (OISST, ref. 26) on a 0.25° grid, high pass filtered using a 5° × 5° boxcar filter. In (**b**), contours indicate SSH (as shown with color shading in (**a**)) with an interval of 0.2 m. All plots are generated with GrADSv2.1.0 (http://cola.gmu.edu/grads/grads.php).

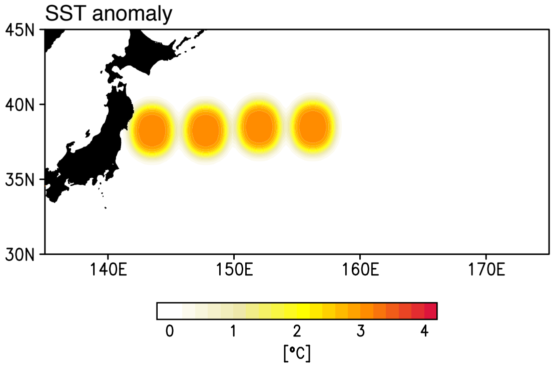

Supplementary Figure. 3. Warm eddies in EDDY run. SST anomalies representing warm mesoscale ocean eddies in EDDY run (see Methods). All plots are generated with GrADSv2.1.0 (http://cola.gmu.edu/grads/grads.php).

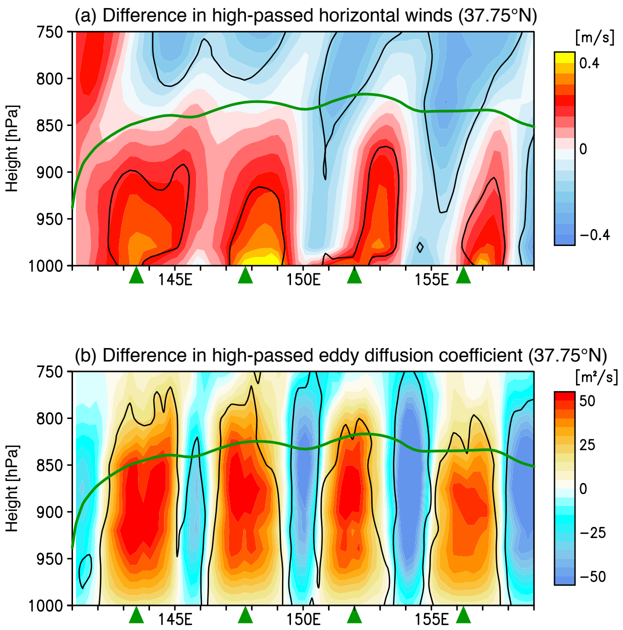

Supplementary Figure 4. Simulated winds and eddy momentum diffusion coefficient response to warm ocean eddies. Height–longitude cross section along 37.75°N of the difference in (**a**) high-pass-filtered winds (m s^{-1}) and (**b**) high-pass-filtered eddy momentum diffusion coefficient (m^2 s^{-1}) between EDDY and CTRL runs. Black solid lines represent statistically significant areas exceeding 90% confidence level. Green triangles at the bottom of each panel indicate latitudinal positions of warm ocean eddy centers in EDDY run. The green line represents the MABL height in EDDY run. All plots are generated with GrADSv2.1.0 (http://cola.gmu.edu/grads/grads.php).

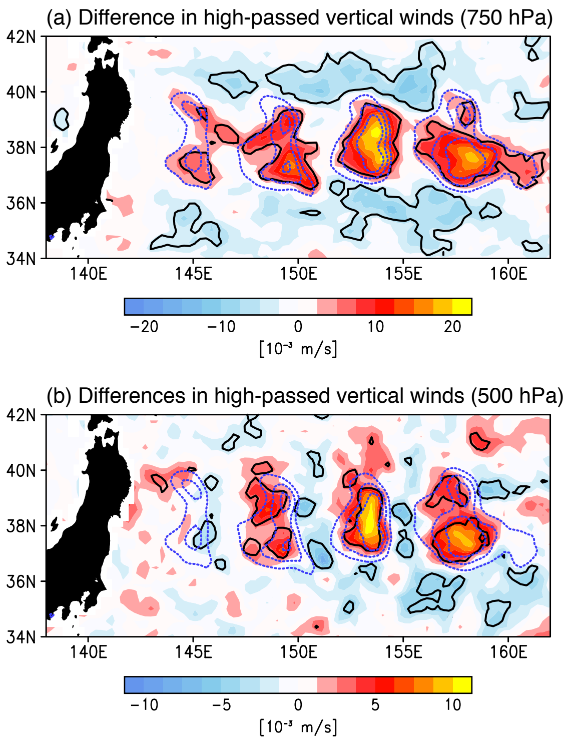

Supplementary Figure 5. Simulated upward vertical wind response to warm ocean eddies. Difference between EDDY and CTRL runs as in Supplementary Fig. 4, but for the horizontal distribution of high-pass-filtered upward vertical winds (10^{-3} m s^{-1}) at (**a**) 750 and (**b**) 500 hPa levels. Black solid lines represent statistically significant regions exceeding 90% confidence level. Dashed contours indicate surface wind convergence (for 3, 6, and 9 × 10^{-6} s^{-1}). All plots are generated with GrADSv2.1.0 (http://cola.gmu.edu/grads/grads.php).

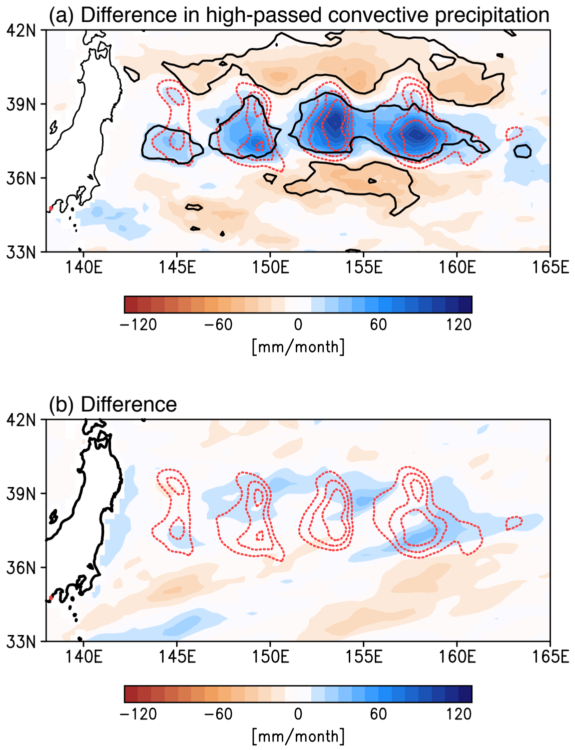

Supplementary Figure 6. Convective precipitation response to warm ocean eddies. Same as Supplementary Fig. 5, but for (**a**) high-pass-filtered convective precipitation (mm month^{-1}) and (**b**) the difference between high-pass-filtered precipitation difference (Fig. 4a) and convective precipitation difference in (**a**). All plots are generated with GrADSv2.1.0 (http://cola.gmu.edu/grads/grads.php).

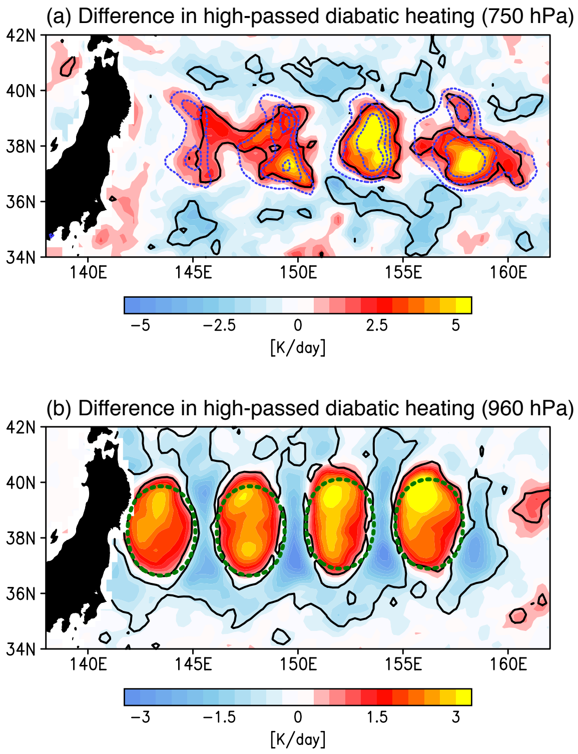

Supplementary Figure 7. Diabatic heating response to warm ocean eddies. Same as Supplementary Fig. 5, but for high-pass-filtered diabatic heating rate (K day^{-1}) at (**a**) 750 and (**b**) 960 hPa levels. In (**b**), dashed lines schematically indicate positions of warm ocean eddies in EDDY run (Supplementary Fig. 3). All plots are generated with GrADSv2.1.0 (http://cola.gmu.edu/grads/grads.php).

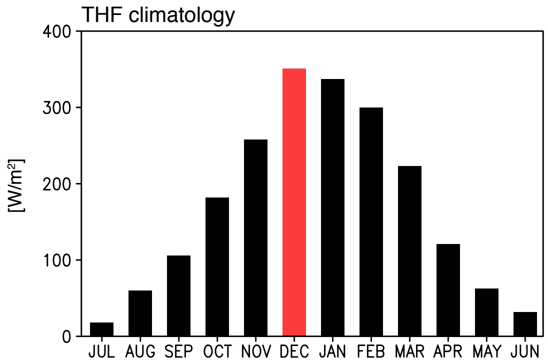

Supplementary Figure 8. Monthly THF climatology over the KOC region. From J-OFURO2. All plots are generated with GrADSv2.1.0 (http://cola.gmu.edu/grads/grads.php).
